# In Memoriam

**Published:** 1896-09

**Authors:** Thomas S. Dunning, Joseph C. Guernsey, Calvin B. Knerr, Carl V. Vischer, Weston D. Bayley


### IN MEMORIAM—DR. CHARLES G. RAUE.

Resolutions adopted by the Homœopathic Medical Society of the County of Philadelphia.

Inasmuch as Dr. Charles G. Raue, one of the oldest homœopathic physicians of this community, has departed from his labors, and, as a sheaf of ripe wheat, has been gathered by the Eternal Reaper;

The Homœopathic Medical Society of the County of Philadelphia adopts this minute:

We realize that a strong man has left us; that a prince has fallen; that scientific medicine has lost an earnest student, a close observer, a careful experimenter, and an enthusiastic advocate. Homœopathy has lost a faithful adherent and one devoted to its deepest truths. His patients have lost a faithful counselor. We, his brother physicians, have lost a cordial friend, a genial companion, and a father in medicine, who was ever ready to lend of his immense store of learning and experience to those who called upon him.

He was one of that strong coterie of men, among whom were Hering, Jeanes, Guernsey, Lippe, Neidhard, Williamson, and Kitchen, who made the practice of Homœopathy respected even when it was weak and new. He bore his part in that battle.

We shall ever remember his genial smile and his cordial greeting.

We extend our sympathy to his family and friends.

We call for a memorial meeting of the profession to do honor to his memory.

A suitably prepared copy of this minute shall be presented to the family of Dr. Raue, and also copies be sent to the press and to the medical journals.

Thomas S. Dunning, M. D.
Joseph C. Guernsey, M. D.
Calvin B. Knerr, M. D.
Carl V. Vischer, M. D.
Weston D. Bayley, M. D.

